# Longitudinal Associations of Serum Orexin‐A With Physical Activity and Sleep in Schizophrenia: A Preliminary Study

**DOI:** 10.1002/npr2.70079

**Published:** 2025-11-30

**Authors:** Akira Tanaka, Yusuke Arai, Takehiko Yasaki, Kentaro Saito, Erika Nakashizuka, Shinsuke Yoshida, Yuka Nakajima, Mika Koido, Kazuhiro Suzuki, Toshinori Nakamura, Daimei Sasayama, Shinsuke Washizuka

**Affiliations:** ^1^ Department of Psychiatry Kurita Hospital Nagano Japan; ^2^ Department of Psychiatry Shinshu University School of Medicine Nagano Japan; ^3^ Department of Community Mental Health Shinshu University School of Medicine Nagano Japan; ^4^ Department of Psychiatry Nagano Red Cross Hospital Nagano Japan

**Keywords:** longitudinal study, orexin‐A, physical activity, schizophrenia, sleep

## Abstract

**Aim:**

Orexin‐A (OXA) is a neuropeptide that regulates arousal, behavior, and sleep–wake rhythms. While altered OXA levels have been observed in schizophrenia, longitudinal data are limited. This study examined the relationship between changes in serum OXA levels and physical activity, sleep, and psychiatric symptoms in patients with chronic schizophrenia.

**Methods:**

Seventeen long‐term inpatients with schizophrenia underwent assessments at baseline (T1) and again after 6 months (T2). Serum OXA levels, sleep and activity parameters derived from actigraphy, and psychiatric symptoms (evaluated with the Positive and Negative Syndrome Scale [PANSS]) were collected at both time points. Spearman's correlations were used to analyze associations between the T2–T1 change in serum OXA (ΔOXA) and the corresponding Δ values of each sleep, activity, and PANSS measure, to explore longitudinal associations among these variables.

**Results:**

ΔOXA was positively correlated with Δ step count (*ρ* = 0.53, *p* = 0.03) and negatively correlated with Δ time in bed (*ρ* = −0.50, *p* = 0.04). No significant associations were found with PANSS scores, other sleep parameters, pharmacotherapy, or body mass index.

**Conclusion:**

In this preliminary longitudinal sample, increases in serum OXA were linked to greater daytime activity and reduced time in bed, suggesting that orexinergic signaling may modulate behavioral regulation in schizophrenia beyond its established role in sleep–wake control. Larger studies, with adjustment for multiple comparisons, are warranted to evaluate OXA as a candidate biomarker or therapeutic target.

## Abstract 2

In this longitudinal study of schizophrenia, increases in serum orexin‐A were associated with greater daytime activity and reduced time in bed, indicating that orexinergic signaling may influence behavioral activation beyond sleep–wake regulation and could serve as a biomarker or therapeutic target.

## Graphical abstract

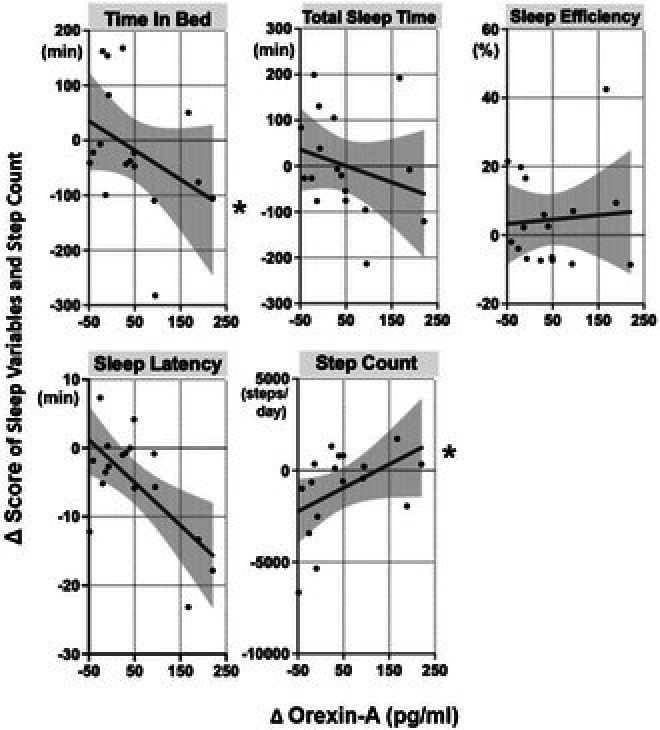

## 1 Introduction

Orexin‐A (OXA) is a neuropeptide involved in the maintenance of wakefulness and the activation of behavior, and its association with psychiatric disorders has attracted increasing attention [1, 2, 3]. OXA originates in the lateral hypothalamus and regulates wakefulness, motivation, physical activity, and sleep rhythm [4, 5, 6]. While OXA levels remain relatively stable in healthy individuals [7, 8], orexin deficiency is a well‐established feature of narcolepsy and is associated with excessive daytime sleepiness, suggesting that dysfunction in this system may contribute to decreased activity and sleep abnormalities [9, 10]. Recently, dysregulation of the orexin system has also been implicated in the pathophysiology of schizophrenia [11].

In schizophrenia, abnormal plasma OXA levels have been reported and are attracting attention in relation to negative symptoms and changes in arousal and activity levels [12, 13]. A proposed hypothesis is that orexin modulates the dopaminergic system via its effects on reward and arousal pathways [14, 15, 16]. In addition, previous reports have indicated that physical activity can transiently elevate orexin levels, while animal studies have demonstrated that sleep deprivation also modulates orexin. These observations suggest that the relationship between orexin, physical activity, and sleep may be bidirectional [17, 18, 19]. Several cross‐sectional studies have reported that plasma OXA levels are lower in patients with schizophrenia compared to healthy controls [12, 20]. Conversely, some studies have reported elevated or unchanged OXA levels, potentially influenced by medication history or body mass index (BMI), highlighting variability in the findings [21, 22, 23].

To date, no studies have directly compared plasma and serum OXA concentrations. Research focusing on serum OXA levels has gradually accumulated within the field of psychiatric disorders. For example, in patients with panic disorder, serum OXA levels were reported to be lower than in healthy controls among drug‐naïve individuals and to significantly increase following therapeutic intervention [24]. In contrast, adolescents with anxiety disorders showed higher serum OXA levels than healthy controls [25], whereas studies on adolescent depression found no group differences or associations with symptom severity [26]. Findings in attention‐deficit/hyperactivity disorder (ADHD) are inconsistent: one study reported decreased serum OXA levels in drug‐naïve children [27], while another found no significant difference between groups [28]. Among women with depressive or anxiety disorders, serum OXA levels did not differ between patients and controls, but were positively correlated with childhood trauma scores [29]. In the field of substance‐use disorders, serum OXA alterations have also been reported: levels were significantly reduced during the acute withdrawal phase in methamphetamine users [30], whereas patients with heroin dependence receiving methadone maintenance therapy exhibited higher levels than healthy controls [31]. Thus, the direction of serum OXA alterations in psychiatric disorders varies depending on disease characteristics, phase, and treatment status, and no consistent trend has emerged. Particularly for schizophrenia, no reports have examined serum OXA; all existing studies have measured plasma OXA levels [12, 13, 20, 21, 22, 23].

Most existing findings are based on cross‐sectional studies, with few longitudinal examinations of the association between OXA and physical activity or sleep parameters [32], and empirical data remain limited regarding whether peripheral OXA levels can serve as functional indicators of central nervous system activity [33].

This study aimed to investigate the longitudinal associations of serum OXA with physical activity, sleep parameters, and negative symptoms in patients with schizophrenia. We evaluated whether the 6‐month change in serum OXA (Δ OXA) was associated with changes in step count, sleep parameters, and negative symptoms in patients with chronic schizophrenia. All participants were hospitalized under conditions with a fixed light–dark cycle (fixed times for lights‐off and lights‐on), minimizing confounding variables. We hypothesized that Δ OXA would be significantly associated with increased physical activity and improvements in sleep efficiency and negative symptoms. Verifying this hypothesis may help elucidate the role of the orexin system in functional outcomes in schizophrenia and contribute to the exploration of its potential as a biomarker or therapeutic target in the future.

## 2 Methods

### 2.1 Study Design and Participants

This single‐center observational study aimed to evaluate the longitudinal associations between serum OXA levels and clinical variables measured using the Positive and Negative Syndrome Scale (PANSS) [34] and actigraphy in patients with chronic schizophrenia undergoing long‐term hospitalization.

The study was conducted at a non‐public psychiatric hospital in Japan. Eligible participants were patients diagnosed with schizophrenia according to the Diagnostic and Statistical Manual of Mental Disorders, Fifth Edition (DSM‐5) [35], by their attending psychiatrists based on medical records. Inclusion criteria were a duration of illness of at least 15 years, age under 65 years, and continuous hospitalization for over 1 year, reflecting the unique Japanese context of long‐term inpatient psychiatric care.

A total of 17 patients were enrolled in the study. At baseline (T1: February–April 2024), all participants underwent PANSS assessments and 1 week of actigraphy to evaluate physical activity and sleep parameters. The same assessments were repeated 6 months later (T2: August–October 2024), while the patients were still hospitalized.

This study was approved by the Ethics Committee of Kurita Hospital (approval no. 202507) and conducted in accordance with the Declaration of Helsinki. All participants received a thorough explanation of the study's purpose, procedures, potential benefits and risks, and data protection measures. Informed consent was obtained in writing and through opt‐out procedures. Only patients whose safety for participation was confirmed by their attending psychiatrists were included.

### 2.2 Procedures and Measures

At T1, demographic and clinical data were collected for each participant. Specifically, background information—including age, sex, age at onset, years of education, duration of illness, and details of pharmacological treatment (types and use of antipsychotics, hypnotics, mood stabilizers, and anticholinergic agents)—was obtained from electronic medical records.

At both T1 and T2, participants underwent assessments using the PANSS and actigraphy‐based evaluation of step count and sleep parameters. Data on physical activity and sleep were continuously recorded for 1 week. The same trained rater administered PANSS at both time points, and total, positive, negative, and general psychopathology subscale scores were calculated.

During the actigraphy assessment period, the use of as‐needed medications was generally prohibited. Participants were instructed to wear the actigraph device continuously each day, except during washing and bathing. Days with less than 80% device wear time were excluded from the analysis. For each valid monitoring period, the average daily step count (SC) and sleep parameters—sleep latency (SL), total sleep time (TST), time in bed (TIB), and sleep efficiency (SE)—were calculated. Sleep and wake states were analyzed using the Cole–Kripke algorithm implemented in ActiLife 6 software (ActiGraph, Pensacola, FL, USA).

Blood samples were collected at both T1 and T2. Venous blood was drawn in the morning (between 8:00 and 10:00 AM) into tubes and immediately placed on ice. Serum was separated by centrifugation, aliquoted, and stored at −60°C until biochemical analysis. Serum OXA levels were measured using a Human OXA ELISA kit (Assay Genie, Dublin, Ireland). All serum samples were assayed in duplicate, and paired samples from each participant (T1 and T2) were analyzed using the same ELISA kit lot to ensure consistency. The intra‐assay coefficients of variation (CVs) ranged from 0.2% to 25.7%. Although we had planned to exclude samples with CVs greater than 30%, no samples met this criterion. Most samples exhibited acceptable variability (< 10%), and therefore, no samples were excluded from the analysis.

### 2.3 Statistical Analysis

Statistical analyses were conducted using SPSS version 30 (IBM Corp.). A two‐tailed *p* < 0.05 was considered statistically significant. However, given the exploratory nature of the study and the absence of adjustments for multiple comparisons, the findings should be interpreted as hypothesis‐generating rather than confirmatory.

Demographic and clinical characteristics at T1 and T2 are summarized in Table 1. The Shapiro–Wilk test indicated that continuous variables were not normally distributed. Therefore, continuous variables are presented as medians with interquartile ranges (Q1–Q3), and categorical variables are presented as percentages.

**TABLE 1:** Demographic and clinical characteristics of participants at T1 and T2.

	T1 median (Q1–Q3) (*n* = 17)	T2 median (Q1–Q3) (*n* = 17)
Age (years)	59.0 (55.0–62.0)	—
Sex, *n* (%)		
Males	11 (64.7)	—
Females	6 (35.3)	—
Age at onset (years)	22.0 (19.0–23.0)	—
Years of education (years)	12.0 (12.0–14.0)	—
BMI (kg/m^2^)	21.3 (18.2–23.4)	21.7 (19.2–24.1)
Medication		
Antipsychotic, *n* (%)	17 (100)	17 (100)
CPZ‐eq dosage (mg/day)	951.5 (750.0–1200.0)	900.0 (600.0–1286.4)
Clozapine, *n* (%)	0 (0)	0 (0)
Mood stabilizer, *n* (%)	7 (41.2)	6 (35.3)
Benzodiazepine, *n* (%)	6 (35.3)	5 (29.4)
Anticholinergic, *n* (%)	5 (29.4)	4 (23.5)
Orexin receptor antagonist, *n* (%)	6 (35.3)	5 (29.4)
Melatonin receptor agonist, *n* (%)	0 (0)	0 (0)
PANSS		
Positive	15.0 (8.0–18.0)	11.0 (9.0–14.0)
Negative	13.0 (7.0–17.0)	9.0 (7.0–13.0)
General	31.0 (20.0–35.0)	21.0 (19.0–26.0)
Total	56.0 (34.0–71.0)	42.0 (36.0–53.0)
Actigraphy variables		
Time in bed (min)	532.8 (424.7–541.8)	489.7 (449.3–517.5)
Total sleep time (min)	371.8 (339.5–443.5)	376.5 (345.2–434.3)
Sleep efficiency (%)	78.8 (69.0–86.9)	80.7 (78.0–84.0)
Sleep latency (min)	5.7 (1.3–8.8)	1.0 (0.0–7.0)
Step counting (steps/day)	6292.8 (40 94.8–11 674.0)	6626.2 (4459.0–8613.8)
Orexin‐A (pg/mL)	392.5 (349.6–423.1)	454.0 (351.9–559.9)

Abbreviations: BMI, body mass index; CPZ‐eq, chlorpromazine equivalents (of antipsychotics); PANSS, Positive and Negative Syndrome Scale.

Subsequently, associations between Δ OXA levels and changes in other clinical or actigraphy‐derived measures were evaluated using Spearman's rank correlation coefficients (Table 2). Pharmacotherapy was considered by examining the correlation between Δ OXA and Δ CPZ‐eq. Patients who were prescribed mood stabilizers, benzodiazepines, anticholinergics, or orexin receptor antagonists at T1 largely continued these medications at T2, with no substantial changes in prescription rates (see Table S1 for detailed prescription status); therefore, their overall influence was considered minimal.

**TABLE 2:** Spearman's rank correlation coefficient between changes in serum OXA and changes in clinical and behavioral variables.

	ΔBMI	ΔCPZ‐eq	Δ PANSS				Δ Actigraphy variables				
			P	N	G	T	TIB	TST	SE	SL	SC
ΔOXA	−0.13	0.10	0.02	−0.28	−0.32	−0.28	**−0.50**	−0.37	−0.22	−0.40	**0.53**

*Note:* *p* < 0.05 are shown in bold.

Abbreviations: BMI, body mass index; CPZ‐eq, chlorpromazine equivalents (of antipsychotics); G, general; N, negative; P, positive; PANSS, Positive and Negative Syndrome Scale; SC, step counting; SE, sleep efficiency; SL, sleep latency; T, total; TIB, time in bed; TST, total sleep time.

However, to further examine the potential effect of orexin receptor antagonists, we additionally conducted a subgroup analysis. Specifically, Spearman's rank correlation analyses between Δ OXA and Δ values of clinical and behavioral variables were performed separately for participants who continued orexin receptor antagonists from T1 to T2 (DORA‐maintained group, *n* = 5) and for those who did not use these agents at either time point (non‐DORA group, *n* = 11) (see Tables S2 and S3).

## 3 Results

### 3.1 Participant Characteristics

Seventeen long‐term inpatients with chronic schizophrenia participated in this study. As shown in Table 1, the median age was 59 years (interquartile range [IQR]: 55–62), and 11 participants (64.7%) were male. The median age at illness onset was 22.0 years (IQR: 19.0–23.0), and the median education was 12 years (IQR: 12.0–14.0). The median BMI was 21.3 (IQR: 18.2–23.4) at T1 and 21.7 (IQR: 19.2–24.1) at T2.

All patients received antipsychotic medications, with a median chlorpromazine‐equivalent (CPZ‐eq) [36] doses of 951.5 mg (IQR: 750.0–1200.0) at T1 and 900.0 mg (IQR: 600.0–1286.4) at T2. No patients were prescribed clozapine. The number of patients receiving mood stabilizers decreased from 7 (41.2%) at T1 to 6 (35.3%) at T2 (−5.9%). Similarly, benzodiazepine use decreased from 6 (35.3%) to 5 (29.4%) (−5.9%), anticholinergic use from 5 (29.4%) to 4 (23.5%) (−5.9%), and orexin receptor antagonist use from 6 (35.3%) to 5 (29.4%) (−5.9%). No patients received melatonin receptor agonists.

In terms of clinical assessment using the PANSS, the median positive score changed from 15 (IQR: 8–18) to 11 (IQR: 9–14), the negative score changed from 13 (IQR: 7–17) to 9 (IQR: 7–13), the general psychopathology score changed from 31 (IQR: 20–35) to 21 (IQR: 19–26), and the PANSS total score changed from 56 (IQR: 34–71) to 42 (IQR: 36–53).

Regarding sleep and physical activity parameters, TIB changed from a median of 532.8 min (IQR: 424.7–541.8) to 489.7 min (IQR: 449.3–517.5), and TST changed from 371.8 min (IQR: 339.5–443.5) to 376.5 min (IQR: 345.2–434.3). SE changed from 78.8% (IQR: 69.0–86.9) to 80.7% (IQR: 78.0–84.0). SL changed from 5.7 min (IQR: 1.3–8.8) to 1.0 min (IQR: 0.0–7.0). The median number of SC per day changed from 6292.8 steps (IQR: 4094.8–11674.0) to 6626.2 steps (IQR: 4459.0–8613.8).

### 3.2 Correlations Between Δ OXA and Changes in Clinical and Behavioral Variables

As shown in Table 2, Spearman's correlation analysis revealed a significant negative correlation between the Δ OXA and the change in TIB (*ρ* = −0.50, *p* = 0.04), and a significant positive correlation with the change in daily step count (Δ SC) (*ρ* = 0.53, *p* = 0.03). No other variables showed significant correlations with Δ OXA.

To further evaluate whether the use of orexin receptor antagonists affected outcomes, we conducted a subgroup analysis. In the DORA‐maintained group (*n* = 5) and the non‐DORA group (*n* = 11), Spearman's correlations between Δ OXA and Δ variables were calculated (Tables S2 and S3).

**FIGURE 1:**
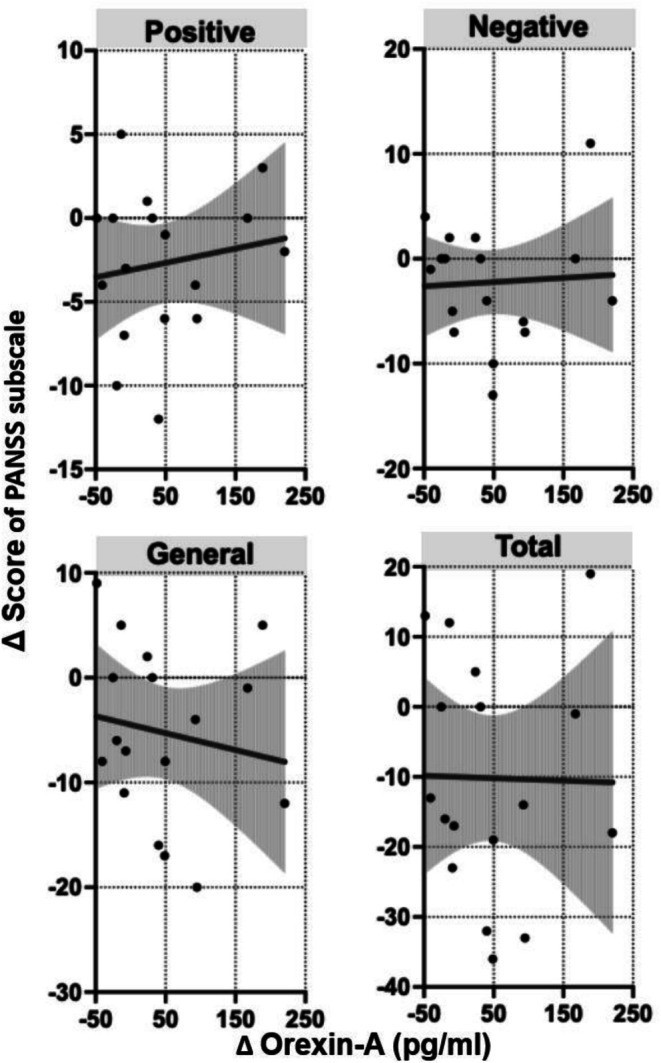
Associations between changes in serum OXA levels (Δ OXA) and changes in PANSS subscale scores. Scatter plots with fitted linear regression lines and 95% confidence intervals (gray shaded areas) illustrate the relationships between Δ OXA (pg/mL) and changes in PANSS Positive (top left), Negative (top right), General (bottom left), and Total (bottom right) scores. No statistically significant associations were observed across subscales.

**FIGURE 2:**
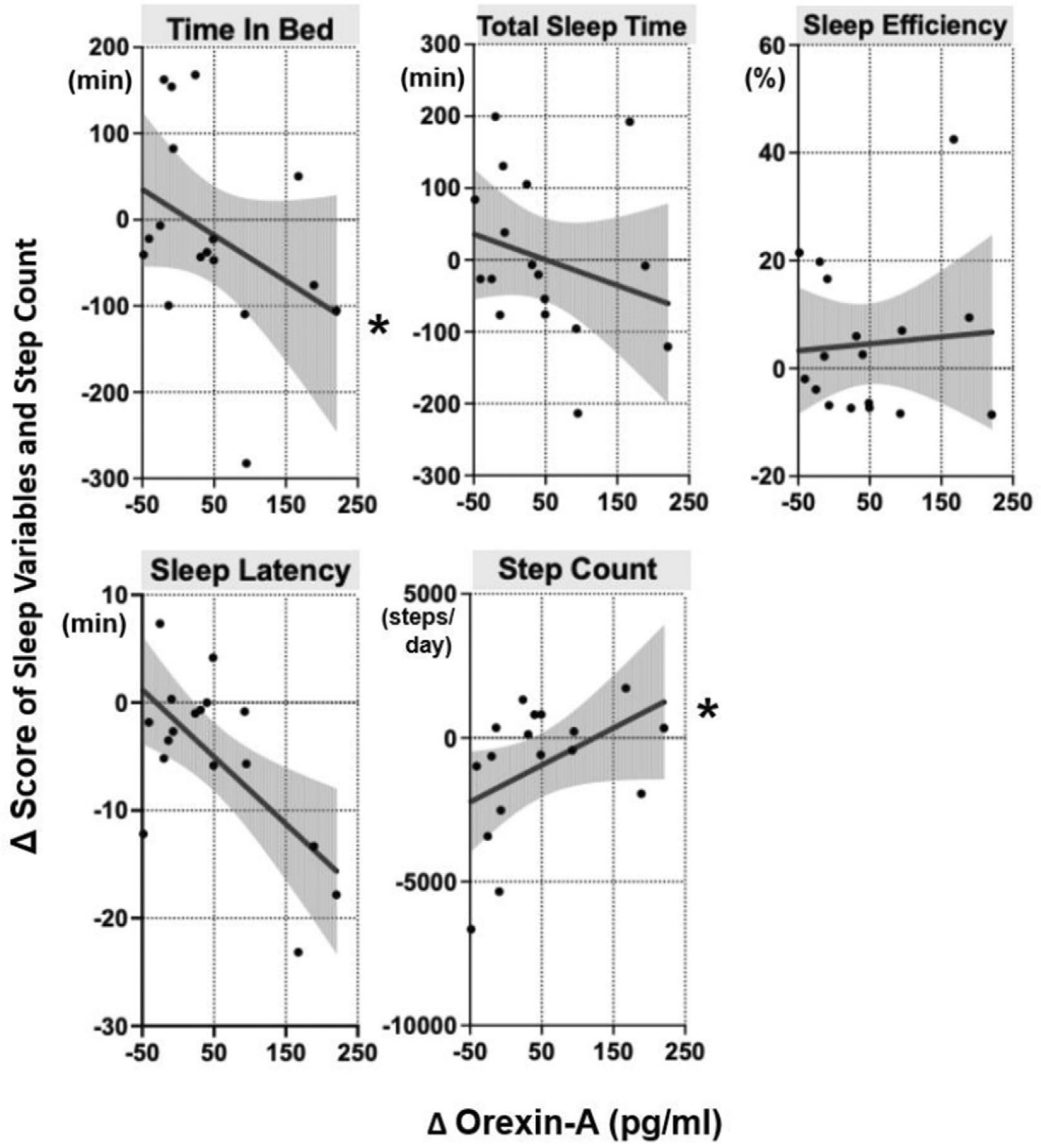
Associations between Δ OXA levels and changes in sleep‐related variables and physical activity. Scatter plots with fitted linear regression lines and 95% confidence intervals (gray shaded areas) depict relationships between Δ OXA (pg/mL) and changes in time in bed, total sleep time, sleep efficiency, sleep latency, and step count. *Statistically significant associations (*p* < 0.05).

Figure 1 presents scatter plots of Δ OXA and the changes in PANSS subscale scores, and Figure 2 illustrates the relationships between Δ OXA and changes in sleep parameters and daily step count.

## 4 Discussion

This longitudinal study investigated chronic inpatients with schizophrenia over 6 months and demonstrated that Δ OXA was significantly associated with increased physical activity (Δ SC: *ρ* = 0.525, *p* = 0.031) and decreased TIB (Δ TIB: *ρ* = −0.495, *p* = 0.043). These findings support the hypothesis that OXA contributes to functional outcomes by modulating both behavioral activation and sleep–wake rhythms. To our knowledge, this is the first report to longitudinally track within‐subject changes in OXA alongside these behavioral indices in patients with schizophrenia.

Clinically, reduced physical activity and prolonged bed rest are critical issues in schizophrenia, as they are linked to negative symptom exacerbation and functional decline. Our findings suggest that interventions targeting the OXA system—such as aerobic exercise programs, behavioral activation therapies, or experimental administration of OXA receptor agonists—may enhance daily activity levels and improve social functioning.

The primary strength of this study lies in its control of confounding factors. All participants were long‐term inpatients in the same ward with uniform schedules for meals, sleep, and medication, minimizing the influence of environmental variability. In addition, the longitudinal design using within‐subject changes allowed for the elimination of interindividual baseline differences. Furthermore, continuous measurement of both activity and sleep using the same actigraphy device yielded objective and high‐frequency data.

Our results are consistent with those of previous cross‐sectional studies that have reported a positive association between OXA and physical activity in healthy individuals [37]. The inverse relationship between OXA and sleep duration aligns with findings in a subset of narcolepsy and in insomnia [38, 39]. Conversely, while prior studies indicated that OXA levels might be affected by medication history or BMI, we did not find any significant associations with these factors (ΔCPZ‐eq: *ρ* = 0.101, *p* = 0.70; ΔBMI: ρ = −0.127, *p* = 0.626). Discrepancies may reflect differences in disease phase, sample characteristics, or symptom assessment methods.

Furthermore, the directions of the observed associations warrant cautious interpretation. Although an increase in serum OXA was accompanied by reduced TIB and increased physical activity, it remains unclear whether changes in OXA contribute to behavioral activation, or conversely, whether enhanced activity and altered sleep patterns stimulate OXA secretion. Previous research provides evidence supporting both interpretations. Several studies have shown that physical activity and sleep deprivation can modify OXA levels, consistent with the notion that behavioral or sleep‐related factors influence OXA dynamics. For instance, a single session of aerobic exercise has been reported to elevate plasma OXA concentrations [17], and animal studies have demonstrated that sleep deprivation alters OXA reactivity [18, 19]. Conversely, extensive evidence indicates that OXA itself promotes arousal and behavioral activation. Neurobiologically, OXA neurons project widely from the lateral hypothalamus to neural systems involved in arousal and reward processing [40, 41, 42]. Moreover, OXA‐deficient mice exhibit reduced activity and disrupted sleep–wake rhythms, underscoring the role of the OXA system in regulating both behavior and sleep [43, 44, 45]. Administration of exogenous OXA increases the firing of locus coeruleus neurons and promotes wakefulness [46]. Systemic OXA administration in narcoleptic dogs reduces cataplexy frequency and stabilizes sleep–wake rhythms [47], while intracerebroventricular infusion of OXA suppresses cataplexy in mice [48]. In addition, the OX2 receptor agonist danavorexton has been shown to dose‐dependently enhance wakefulness in both humans and animals [49]. Taken together, these findings suggest that OXA and the regulation of activity and sleep are bidirectionally related and may influence each other. Future studies employing high–temporal‐resolution longitudinal assessments are needed to clarify the causal relationship between changes in OXA and behavioral alterations. In particular, to establish OXA as a biomarker of behavioral activation and daytime functioning, it is essential to determine which direction of change more strongly predicts clinical outcomes. Overall, the present results support the concept of an “OXA–behavioral rhythm modulation hypothesis,” although further research is warranted to elucidate how peripheral OXA concentrations reflect central activity and how these changes relate to symptom improvement.

In this study, all participants were long‐term inpatients residing in the same ward, where environmental factors such as lighting, bedtimes and wake times, and meal schedules were standardized. Nevertheless, serum OXA levels exhibited changes over the 6‐month observation period. These changes may have been influenced by factors other than variations in SC or TIB.

In healthy individuals, OXA levels show minimal diurnal variation and remain relatively stable throughout the day [7, 8]. However, no studies have examined seasonal fluctuations in OXA among healthy populations, and this aspect remains unclear. In contrast, a study of patients with hypersomnia reported that cerebrospinal fluid OXA levels were higher in summer and positively correlated with daylight duration [50]. Taken together, it is possible that seasonal variation in orexin secretion partially contributed to the changes in serum OXA observed in the present study.

Furthermore, clinical interventions—such as sleep hygiene guidance provided by attending psychiatrists, occupational therapy, and daytime activity programs within the ward—may have indirectly promoted behavioral activation, thereby influencing orexin dynamics. These factors could not be fully controlled within the present study design. Future studies should standardize and document seasonal factors and clinical interventions to enable more rigorous analyses.

This study has several limitations. First, because the sample consisted exclusively of chronic, long‐term inpatients, the external generalizability of the findings is limited. Second, the small sample size may have resulted in insufficient statistical power to detect smaller effects. Third, peripheral serum OXA levels may not accurately reflect central nervous system activity, raising questions about the validity of this biomarker. Fourth, sleep architecture was inferred solely from actigraphy, which does not capture detailed sleep stages. Finally, as this was an exploratory pilot study, we did not perform multivariate analyses and instead focused on correlation‐based approaches.

Future studies should involve larger, multicenter longitudinal cohorts including various illness phases and ward environments to confirm replicability and control for confounders. To test causal direction between OXA and activity/sleep, high‐frequency OXA and actigraphy, and time‐lagged and mediation analyses should be employed to establish temporal precedence. Furthermore, combining serum and cerebrospinal fluid OXA measurements with neuroimaging and polysomnographic analysis may help elucidate central–peripheral OXA dynamics and detailed sleep architecture.

In conclusion, this preliminary longitudinal study demonstrated that increased serum OXA was associated with enhanced physical activity and reduced sleep duration among patients with chronic schizophrenia. The findings suggest that the OXA system may contribute to functional outcomes by modulating both behavioral activation and the sleep–wake cycle. Future large‐scale studies with rigorous control of confounding variables and interventional trials are warranted to assess the therapeutic potential and generalizability of OXA‐targeted strategies in schizophrenia.

## Author Contributions

Akira Tanaka and Yusuke Arai contributed to the manuscript conception. Takehiko Yasaki, Kentaro Saito, Erika Nakashizuka, Shinsuke Yoshida, Yuka Nakajima, Mika Koido, Kazuhiro Suzuki, Toshinori Nakamura, Daimei Sasayama, and Shinsuke Washizuka reviewed the manuscript. Akira Tanaka and Yusuke Arai wrote the first draft of this manuscript. Takehiko Yasaki, Kentaro Saito, Erika Nakashizuka, Shinsuke Yoshida, Yuka Nakajima, Mika Koido, Kazuhiro Suzuki, Toshinori Nakamura, Daimei Sasayama, and Shinsuke Washizuka wrote the manuscript. Akira Tanaka, Yusuke Arai, Yuka Nakajima, and Mika Koido obtained consent, conducted interviews, and performed examinations for research. Akira Tanaka and Yusuke Arai conducted all statistical analyses. All authors contributed to the manuscript revision and have read and approved the submitted version.

## Funding

The authors have nothing to report.

## Disclosure

*Registry and the Registration No. of the Study/Trial*: Trial registration: Longitudinal associations between serum orexin A levels and physical activity, sleep, and clinical indicators in patients with schizophrenia: an exploratory study (UMINID: 000058727), registered on August 7, 2025.

*Research Involving Human Participants*: All procedures involving human participants were conducted in accordance with the ethical standards of the institutional and national research committees and the 1964 Declaration of Helsinki and its later amendments or comparable ethical standards.

## Ethics Statement

This study protocol was approved by the Ethics Committee of Kurita Hospital (approval no. 202507) and conducted in accordance with the Declaration of Helsinki.

## Consent

This study was conducted using an opt‐out consent process approved by the institutional ethics committee.

## Conflicts of Interest

The authors declare no conflicts of interest.

## Supporting information


**Table S1:** Raw data at baseline (T1) and at 6 months (T2), including demographic characteristics, clinical measures, sleep/activity parameters, and serum orexin‐A levels.

**Table S2:** Spearman's rank correlation coefficients between changes in serum OXA and changes in clinical and behavioral variables in the DORA‐maintained group (*n* = 5).
**Table S3:** Spearman's rank correlation coefficients between changes in serum OXA and changes in clinical and behavioral variables in the non‐DORA group (*n* = 11).

## Data Availability

The data that support the findings of this study is available in the Supporting Information of this article.
