# Supplementary material for: Longitudinal Associations of Serum Orexin‐A With Physical Activity and Sleep in Schizophrenia: A Preliminary Study
Source: Neuropsychopharmacol Rep. 2025 Nov 30;45(4):e70079. doi: 10.1002/npr2.70079 (PMC12665116; doi:10.1002/npr2.70079)
Supplement: Supplementary file 2 — Table S2: Spearman's rank correlation coefficients between changes in serum OXA and changes in clinical and behavioral variables in the DORA‐maintained group (n = 5). Table S3: Spearman's rank correlation coefficients between changes in serum OXA and changes in clinical and behavioral variables in the non‐DORA group (n = 11). [file NPR2-45-e70079-s001.docx]

| **Table S2.** Spearman’s rank correlation coefficients between changes in serum OXA and changes in clinical and behavioral variables in the DORA-maintained group (n = 5). | | |
| --- | --- | --- |
|  |  |  |
| **Variable** | **Spearman’s rho** | **p value** |
| ΔBMI | −0.60 | 0.29 |
| ΔCPZ-eq | 0.40 | 0.51 |
| ΔPANSS Positive | −0.10 | 0.87 |
| ΔPANSS Negative | −0.50 | 0.39 |
| ΔPANSS General | 0.30 | 0.62 |
| ΔPANSS Total | −0.30 | 0.62 |
| ΔTIB | −0.70 | 0.19 |
| ΔTST | −0.40 | 0.51 |
| ΔSE | −0.50 | 0.39 |
| ΔSL | 0.10 | 0.87 |
| ΔSC | 0.60 | 0.29 |
|  |  |  |
| Abbreviations: BMI, body mass index; CPZ-eq, chlorpromazine equivalents (of antipsychotics); PANSS, Positive and Negative Syndrome Scale; TIB, time in bed; TST, total sleep time; SE, sleep efficiency; SL, sleep latency; SC, step counting. | | |

| **Table S3.** Spearman’s rank correlation coefficients between changes in serum OXA and changes in clinical and behavioral variables in the non-DORA group (n = 11). | | |
| --- | --- | --- |
|  |  |  |
| **Variable** | **Spearman’s rho** | **p value** |
| ΔBMI | 0.00 | 1.00 |
| ΔCPZ-eq | −0.23 | 0.51 |
| ΔPANSS Positive | −0.01 | 0.97 |
| ΔPANSS Negative | −0.27 | 0.43 |
| ΔPANSS General | −0.42 | 0.19 |
| ΔPANSS Total | −0.29 | 0.39 |
| ΔTIB | −0.58 | 0.06 |
| ΔTST | −0.45 | 0.17 |
| ΔSE | −0.18 | 0.59 |
| ΔSL | **−0.62** | **0.04** |
| ΔSC | 0.52 | 0.10 |
|  |  |  |
| Abbreviations: BMI, body mass index; CPZ-eq, chlorpromazine equivalents (of antipsychotics); PANSS, Positive and Negative Syndrome Scale; TIB, time in bed; TST, total sleep time; SE, sleep efficiency; SL, sleep latency; SC, step counting. P-values <0.05 are shown in **bold**. | | |
